# GeoSeqNet: A Geometry-Aware Sequential Network for Robust 3D Point Cloud Analysis

**DOI:** 10.3390/s26144511

**Published:** 2026-07-16

**Authors:** Dongzhen Liu, Yuzhong Deng, Haojie Wu, Jianxiao Zou, Shicai Fan

**Affiliations:** 1 School of Automation Engineering, University of Electronic Science and Technology of China, Chengdu 611731, China; dongzhenliu0404@std.uestc.edu.cn (D.L.); yuzhong.deng@mail.mcgill.ca (Y.D.); musk@std.uestc.edu.cn (H.W.); jxzou@uestc.edu.cn (J.Z.); 2 Shenzhen Institute for Advanced Study, University of Electronic Science and Technology of China, Shenzhen 518110, China

**Keywords:** 3D point cloud analysis, geometry-aware learning, long-range contextual interaction, sequential modeling, robust point cloud understanding

## Abstract

3D point cloud understanding plays a vital role in remote sensing, robotic perception and intelligent scene analysis. However, real-world point cloud data are often affected by sensing noise, incomplete geometry, occlusion, and irregular sampling, posing significant challenges to reliable geometric representation learning and long-range contextual modeling. Existing methods typically rely on fixed neighborhood aggregation or computationally expensive global interaction mechanisms, leaving considerable room for improvement in terms of robustness and efficiency under complex sensing conditions. To address these challenges, we propose GeoSeqNet, a geometry-aware contextual learning framework for robust 3D point cloud analysis. Specifically, an Enhanced Local Operator (ELO) is introduced to strengthen local geometric representation, while a Geometric Encoding Module (GEM) is employed to preserve spatial geometric information during long-range feature interactions. In addition, an Adaptive Gate Fusion (AGF) module is designed to effectively integrate Gate-Scaled LSTM and GRU branches, enabling efficient long-range contextual modeling. By jointly exploiting local geometric cues and long-range contextual information, GeoSeqNet achieves robust feature learning with low computational overhead. Extensive experiments on ModelNet40, ScanObjectNN, and ShapeNetPart demonstrate the effectiveness of GeoSeqNet. The proposed method achieves competitive performance while maintaining a favorable efficiency–accuracy trade-off and exhibits strong robustness in complex real-world scenarios. These results indicate that GeoSeqNet provides an effective and reliable solution for point cloud understanding in challenging sensing environments.

## 1. Introduction

3D point cloud analysis is a fundamental problem in computer vision and robotics, with broad applications in remote sensing, autonomous driving, robotic perception, augmented reality, and industrial inspection [1,2,3,4]. Unlike images and videos defined on regular grids, point clouds consist of irregular and unordered 3D samples that directly preserve geometric and spatial structures of real-world environments. Such characteristics provide rich spatial representation capability while also making robust point cloud understanding highly challenging under complex sensing conditions.

In real-world scenarios, point cloud data are often affected by noise, occlusion, incomplete geometry, and irregular sampling. Therefore, an effective point cloud model should not only capture fine-grained local geometric structures but also establish robust contextual relationships across spatially distant regions. Early point cloud learning methods, represented by PointNet [5] and its variants [6,7], treat point clouds as unordered sets and achieve permutation invariance through symmetric aggregation functions. Although these methods demonstrate effectiveness in global representation learning, their capability for local geometric modeling remains limited. To alleviate this issue, PointNet++ [6] and subsequent approaches introduced hierarchical sampling and neighborhood grouping mechanisms to improve local structure learning [8,9,10,11]. More recently, graph-based interaction methods and geometry-aware convolution operators further enhanced local geometric representation through dynamic graph construction and adaptive neighborhood aggregation.

Despite notable progress in local geometric modeling, most existing approaches still mainly rely on local neighborhood aggregation and short-range feature interaction. As a result, establishing robust long-range contextual relationships under complex geometric environments remains challenging. To address this issue, Transformer-based architectures [12,13,14,15] have recently attracted considerable attention in point cloud analysis. By introducing self-attention mechanisms, these methods can explicitly capture global dependencies among point features. However, self-attention operations usually incur quadratic computational and memory complexity with respect to the number of points, limiting their efficiency in large-scale point cloud analysis. In addition, similarity-based attention mechanisms often exhibit limited robustness under noisy observations, incomplete geometry, and irregular sampling conditions [16]. Sequential modeling provides an alternative paradigm for long-range dependency learning by propagating contextual information along ordered feature representations, offering lower computational complexity than self-attention-based approaches.

Although existing methods have introduced geometric information into local operators, attention mechanisms, or sequence construction strategies, how to consistently preserve and coordinate explicit spatial geometric context during efficient long-range feature interaction remains insufficiently explored. In real-world point clouds, spatial geometric information may gradually weaken as feature interaction becomes increasingly dominated by abstract semantic representations. Preserving geometric sensitivity during contextual learning is therefore important for robust point cloud understanding under complex sensing environments.

Meanwhile, although point clouds are inherently unordered, sequential modeling has recently emerged as an efficient alternative for long-range contextual learning. Instead of explicitly modeling all pairwise feature interactions, sequential modeling establishes contextual dependencies through progressive information propagation, thereby significantly reducing computational overhead. Such a property is particularly attractive for large-scale point cloud analysis, where efficient long-range contextual interaction is required under limited computational budgets. Motivated by these observations, we propose GeoSeqNet, a geometry-aware contextual learning framework for robust 3D point cloud analysis. Unlike existing hybrid frameworks that mainly combine local feature extraction with global attention-, sequential-, or SSM-based modeling, GeoSeqNet constructs geometry-enhanced representations and preserves explicit spatial geometric cues during lightweight sequential contextual interaction. Specifically, the proposed framework tightly integrates local geometric feature refinement, spatial geometric context supplementation, and efficient contextual interaction within a unified architecture.

At the local level, an Enhanced Local Operator (ELO) is introduced to refine neighborhood feature responses through selective local weighting, thereby suppressing redundant local interference and improving robustness under noisy and irregular point cloud conditions. To further preserve spatial geometric context during long-range feature interaction, a lightweight Geometric Encoding Module (GEM) is proposed to supplement geometric information during contextual learning. Different from existing geometry-aware methods that mainly incorporate geometric information during local feature extraction or attention weighting, the proposed GEM reintroduces spatial geometric context during long-range feature interaction. Furthermore, an Adaptive Gate Fusion (AGF) module collaboratively integrates Gate-Scaled LSTM (GSLSTM) and GRU branches to achieve efficient long-range contextual learning with relatively low computational overhead.

We conduct extensive evaluations on several mainstream benchmarks, including ModelNet40, ScanObjectNN, and ShapeNetPart. Experimental results demonstrate that GeoSeqNet achieves competitive performance while maintaining favorable efficiency–accuracy trade-offs on both classification and part segmentation tasks. Notably, GeoSeqNet achieves an overall accuracy (OA) of 88.8% on the challenging ScanObjectNN benchmark, demonstrating strong robustness under noisy observations, incomplete geometry, and irregular sampling conditions.

The main contributions of this work are summarized as follows:

1. We propose GeoSeqNet, a unified geometry-aware contextual learning framework for robust 3D point cloud analysis, which integrates local geometric refinement, spatial context supplementation, and efficient long-range contextual propagation within a progressive information flow.
2. We design an Enhanced Local Operator (ELO) and a lightweight Geometric Encoding Module (GEM) to improve local feature robustness and preserve explicit spatial geometric context during long-range feature interaction.
3. We develop an Adaptive Gate Fusion module that collaboratively integrates Gate-Scaled LSTM and GRU branches for efficient long-range contextual learning, enabling favorable efficiency–accuracy trade-offs on multiple point cloud benchmarks.

## 2. Related Work

### 2.1. Point-Based Deep Learning Methods

Point-based deep learning methods constitute a fundamental foundation for 3D point cloud analysis [5,17,18,19]. PointNet [5] pioneered a network architecture that directly processes unordered point sets. By adopting symmetric aggregation functions, it guarantees permutation invariance with a simple and computationally efficient design. However, it is limited in capturing local geometric relationships and fine-grained structural patterns. PointNet++ [6] subsequently introduced hierarchical sampling and multi-scale neighborhood grouping, enabling the network to learn local geometric patterns in complex scenes and significantly improving performance. Building upon this line of research, DGCNN [20] explicitly models local topology by dynamically constructing graphs and applying edge convolutions. Methods such as KPConv [19] and PAConv [21] further enhance local geometry modeling by leveraging learnable or adaptive convolution kernels. CurveNet [22] attempts to incorporate curve structures to better approximate geometric patterns on continuous surfaces. More recently, PointMLP [23] and PointNeXt [11] improve point cloud representation by optimizing MLP architectures and feature reordering strategies, without explicitly constructing neighborhood graphs. Despite notable progress in local geometric modeling, these methods still fundamentally rely on discrete neighborhood partitioning and local aggregation operators, primarily focusing on short-range geometric interactions. Moreover, the strict permutation-invariance assumption makes it difficult to capture long-range structural dependencies across distant regions and global contextual relationships in complex shapes. This limitation suggests that local geometric modeling alone is insufficient for comprehensive understanding of point cloud structures.

### 2.2. Long-Range Contextual Modeling: Self-Attention and State Space Models

To address the limitations of local methods in modeling global structures, researchers have introduced mechanisms for global dependency modeling. The success of Transformer architectures in natural language processing has greatly stimulated their adoption in point cloud learning. Methods such as PCT [12], Point Transformer [24], PTv2 [25], and SAPFormer [26] employ self-attention to explicitly model long-range interactions among points, and further incorporate geometric relations or relative positional encoding, achieving substantial improvements on both classification and segmentation tasks. Meanwhile, self-supervised and pretraining-based approaches have also been widely explored to improve the generalization capability of point cloud representations. For example, Point-BERT [27] and Point-MAE [28] learn transferable representations through masked point modeling, demonstrating the effectiveness of large-scale pretraining for point cloud understanding.

Despite their effectiveness, self-attention mechanisms incur quadratic computational and memory complexity with respect to the number of points, often leading to severe efficiency bottlenecks for high-resolution point clouds or real-time applications. In addition, their pairwise similarity-based modeling paradigm may not sufficiently enforce geometric continuity, making them less robust under noise, occlusions, and non-uniform sampling.

Recently, state space models (SSMs) have emerged as an alternative direction for efficient long-range dependency modeling. PointMamba [29], Mamba3D [30], LFE-PointMamba [31], and SI-Mamba [32] introduce selective SSMs into point cloud learning, enabling global dependency modeling with near-linear complexity and achieving a favorable balance between efficiency and performance. Among them, SI-Mamba [32] further incorporates spectral information into Mamba-based point cloud modeling, enhancing the preservation of geometric relationships through structure-aware sequence construction. Nevertheless, existing SSM-based methods generally introduce geometric information through token ordering, scanning paths, positional encoding, or local aggregation. How to continuously preserve and coordinate such geometric cues during subsequent long-range contextual interaction remains less explicitly explored. Therefore, how to maintain computational efficiency while aligning global dependency modeling more consistently with point cloud geometry remains an open challenge.

### 2.3. Geometric Priors in Point Cloud Learning

Geometric priors have long been recognized as an important factor for improving the robustness and generalization of point cloud models [33]. Early methods employed handcrafted geometric descriptors (e.g., PFH, FPFH, and SHOT) [34,35,36] to characterize local normals and curvature information, but their representational capacity and scalability were limited. With the development of deep learning, researchers began to embed geometric structures into learnable operators. For instance, KPConv [19] explicitly encodes geometric relations within local neighborhoods via kernel point convolution, thereby strengthening the modeling of local spatial structures. In recent years, geometric priors have also been explored from the perspective of structural constraints. A representative direction is to introduce equivariance constraints such as SO(3)/SE(3) [37] into network architectures, ensuring that feature representations remain consistent under rotations or rigid transformations, thus improving robustness to pose variations and spatial perturbations. Such geometry-consistent modeling has been validated in tasks like point cloud registration and representation learning. Examples include SE3ET [38], Equi-GSPR [39], and GETr [40]. Meanwhile, recent studies have attempted to enhance spatial perception by explicitly incorporating geometric information, such as learning-based geometric encodings or equivariant representations that integrate geometric priors directly into feature learning [15,19,41]. Nevertheless, geometric priors in existing methods are predominantly used for local operator design or structural consistency regularization, and are often not tightly coordinated with global structure modeling mechanisms.

In addition, recent studies have also explored robustness enhancement for noisy, distorted, or complex LiDAR point clouds [42] improved the robustness of point cloud analysis through perturbation simulation and distortion-guided feature augmentation, while [43] adopted a multi-task learning framework for robust preprocessing of complex LiDAR point clouds. These studies mainly enhance robustness from the perspectives of data perturbation modeling, feature augmentation, or preprocessing optimization, providing useful insights for stable point cloud modeling under complex conditions. In summary, although substantial progress has been made in local geometry modeling, geometric prior encoding, geometric consistency constraints, and robustness enhancement, these capabilities are still largely introduced as relatively independent components in existing methods. The lack of a unified information-flow framework that coherently integrates geometric priors with efficient global dependency modeling continues to limit the ability to capture long-range structural relationships in complex scenes. This observation motivates the exploration of a unified approach that can jointly integrate geometric priors and efficient global modeling mechanisms within a single framework.

## 3. Proposed Method

### 3.1. Overview of GeoSeqNet

**Figure 1:**
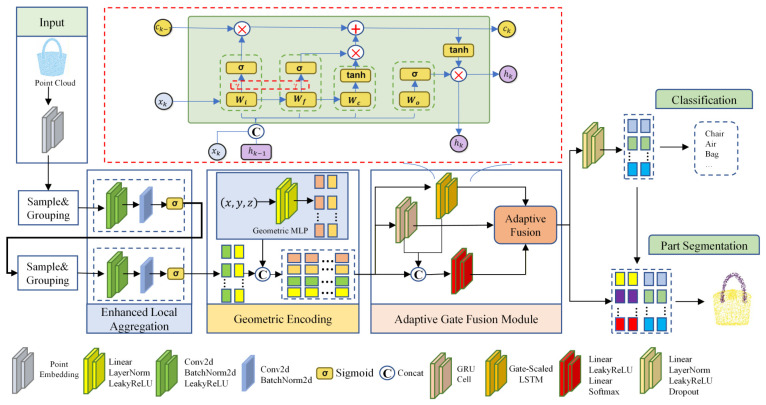
Overall architecture of GeoSeqNet. The framework consists of three key components: Enhanced Local Aggregation for local feature extraction, Geometric Encoding for explicit spatial prior injection, and Adaptive Gate Fusion module for joint long- and short-range dependency learning. The learned features are used for both classification and part segmentation.

The overall architecture of GeoSeqNet is illustrated in Figure 1. GeoSeqNet is a geometry-aware contextual learning framework designed for robust and efficient point cloud analysis under complex sensing environments. The proposed framework organizes local geometric refinement, explicit spatial context supplementation, and long-range contextual propagation into a progressive information flow. It mainly consists of three components: Enhanced Local Operator (ELO), Geometric Encoding Module (GEM), and Adaptive Gate Fusion (AGF). Specifically, ELO refines unreliable neighborhood responses through selective local weighting, thereby improving the robustness of local geometric representation under noisy and irregular point cloud conditions. GEM further supplements coordinate-aware geometric context before long-range feature interaction, which helps preserve the spatial structure of point clouds during contextual modeling. AGF performs efficient contextual interaction through collaborative fusion between Gate-Scaled LSTM and GRU branches, enabling complementary dependency modeling with relatively low computational overhead. Through this coordinated design, GeoSeqNet captures both fine-grained local geometric structures and cross-region contextual dependencies. The learned representations are finally aggregated for downstream tasks such as classification and part segmentation.

### 3.2. Enhanced Local Operator

In practical sensing environments, local neighborhoods may be affected by noisy observations, incomplete geometry, and irregular sampling patterns. As a result, direct neighborhood aggregation may introduce unstable local responses and reduce the robustness of local geometric representations. To address this issue, we propose an ELO, which refines neighborhood responses before local aggregation through a lightweight spatial weighting mechanism.

Unlike attention-based neighborhood aggregation methods that explicitly model pairwise relations among neighboring points, ELO performs reliability-aware neighborhood response refinement before local pooling. Existing attention pooling mechanisms, such as those used in Point Transformer [24], or edge-attention variants related to DGCNN-style neighborhood modelingcite [20], usually assign aggregation weights according to point-to-point relations or edge feature correlations. In contrast, ELO does not formulate neighborhood aggregation as a pairwise attention process. Instead, after local feature transformation, a lightweight spatial weighting branch generates response-level modulation weights for different neighboring positions, which are then applied to neighborhood features before max pooling. In this way, ELO can selectively weaken unstable neighborhood responses while preserving discriminative local geometric cues, thereby providing a simple and effective feature refinement mechanism for irregular and noisy local regions.

Given an input point cloud

(1) $$P={p_{i}}_{i=1}^{N_{p}},\;p_{i}\in R^{3},$$

hierarchical sampling and neighborhood grouping are first performed to construct local regions around sampled centers. The grouped local feature tensor is represented as

(2) $$X\in R^{B\times G\times S\times C},$$

where *B* denotes the batch size, *G* denotes the number of sampled local regions, *S* represents the number of neighboring points in each local region, and *C* denotes the input feature dimension.

Consistent with the implementation, the grouped tensor is first permuted into

(3) $$\bar{X}\in R^{B\times C\times G\times S}.$$

Then, two shared 1×1 convolution layers are used for local feature transformation:

(4) $$F=\phi_{2}\left(\phi_{1}\left(\bar{X}\right)\right),$$

where $\phi_{1}$ and $\phi_{2}$ denote feature transformation blocks consisting of 1×1 convolution, Batch Normalization, and LeakyReLU activation. The transformed local feature representation is given by

(5) $$F\in R^{B\times C^{\prime }\times G\times S},$$

where $C^{\prime }$ denotes the output feature dimension.

To adaptively refine neighborhood responses, a lightweight spatial weighting branch is introduced:

(6) $$W=\sigma ({\mathrm{Conv}}_{1\times 1}\left(F\right)),$$

where

(7) $$W\in R^{B\times 1\times G\times S},$$

and σ(·) denotes the sigmoid activation function. The weighted neighborhood responses are computed as

(8) $$\tilde{F}=F⊙W,$$

where ⊙ denotes element-wise multiplication, and *W* is broadcast along the channel dimension. This operation is consistent with the implementation, where the learned spatial weights are applied to the transformed neighborhood features before aggregation.

Max pooling is then performed along the neighborhood dimension:

(9) $$H_{b,c,g}^{\prime }=\max_{1\leq s\leq S}{\tilde{F}}_{b,c,g,s},$$

where $H^{\prime }\in R^{B\times C^{\prime }\times G}$. Here, *b*, *c*, *g*, and *s* index the batch, channel, local region, and neighboring point dimensions, respectively. Finally, the local feature representation is rearranged as $H\in R^{B\times G\times C^{\prime }}$.

The spatial weighting mechanism is intended to refine unstable neighborhood responses caused by uneven sampling, noise, outliers, or incomplete local structures. In these cases, direct max pooling may retain strong but less reliable responses, thereby affecting the stability of local feature aggregation. By introducing learnable weights before pooling, ELO adaptively adjusts the contributions of different neighboring positions and reduces the influence of unreliable responses. This response refinement process helps provide more stable local geometric representations for subsequent contextual modeling.

### 3.3. Geometric Encoding Module

Although local aggregation effectively captures neighborhood geometric structures, spatial geometric context may gradually weaken as features are progressively abstracted and propagated through long-range contextual interactions. Preserving geometry-awareness during contextual learning is therefore critical for robust point cloud understanding under complex sensing environments. Existing positional encoding strategies are typically introduced at the feature initialization stage to provide spatial cues for subsequent feature interaction. In contrast, the proposed Geometric Encoding Module (GEM) is not intended to encode positional information for initialization. Instead, GEM explicitly reintroduces coordinate-aware geometric context during the contextual learning stage, enabling geometric information to be continuously preserved throughout long-range feature propagation.

Let $Q\in R^{B\times M\times 3}$ denote the coordinates of sampled centers, and $H\in R^{B\times M\times C^{\prime }}$ denote the locally refined features generated by ELO. A lightweight coordinate encoder composed of two linear layers is employed:

(10) $$G=\psi_{2}\left(\psi_{1}\left(Q\right)\right),$$

where $\psi_{1}$ and $\psi_{2}$ denote transformation blocks consisting of Linear, LayerNorm, and LeakyReLU operations. The resulting geometric representation is

(11) $$G\in R^{B\times M\times C_{g}}.$$

The geometric representation is fused with local features through channel-wise concatenation:

(12) $$\hat{H}=\mathrm{Concat}\left(H,G\right),$$

yielding

(13) $$\hat{H}\in R^{B\times M\times (C^{\prime }+C_{g})}.$$

Unlike conventional positional encoding methods that mainly provide spatial awareness during feature initialization, GEM continuously injects coordinate-aware geometric context into the contextual learning process. This design alleviates the progressive loss of geometric information during feature abstraction and long-range propagation, thereby improving robustness under noisy observations, incomplete geometry, and irregular sampling conditions. It should be noted that GEM serves as a geometry context preservation mechanism rather than a conventional positional encoding strategy. While positional encoding primarily facilitates spatially aware feature initialization, GEM focuses on maintaining geometric consistency during long-range contextual interaction.

### 3.4. Adaptive Gate Fusion Module

Given the geometry-enhanced token representation produced by GEM,

(14) $$\hat{H}={\left\{{\hat{h}}_{k}\right\}}_{k=1}^{M},\;{\hat{h}}_{k}\in R^{D_{s}},$$

where *M* denotes the number of sampled local regions and ${\hat{h}}_{k}$ represents the geometry-enhanced feature of the *k*-th local region. The token dimension is defined as

(15) $$D_{s}=C+C_{g}.$$

where *C* denotes the channel dimension of the local feature representation, and $C_{g}$ denotes the channel dimension of the coordinate-aware geometric encoding feature generated by GEM. Each token corresponds to a geometry-enhanced local region obtained through hierarchical sampling, neighborhood aggregation, and geometric encoding, and the resulting token sequence serves as the input to the subsequent sequential contextual learning module. Therefore, the sequence used in GeoSeqNet is not constructed from arbitrary permutations of raw points, but from structured local region representations with explicit geometric context. Based on these geometry-enhanced tokens, we further design an Adaptive Gate Fusion (AGF) module to model long-range dependencies across local regions in the point cloud sequence. Instead of relying on self-attention to explicitly construct global pairwise interactions, AGF performs sequential dependency learning through progressive contextual propagation and adaptive multi-source fusion. Its overall architecture consists of three components: a Gate-Scaled LSTM branch for long-range context accumulation, a GRU branch for complementary compact contextual transition modeling, and a lightweight gating network for adaptive fusion of multiple information streams.

The motivation behind using both Gate-Scaled LSTM and GRU branches is to exploit their complementary modeling characteristics rather than simply increasing the model capacity. The Gate-Scaled LSTM branch introduces an explicit memory cell and a gate-scaled state updating mechanism, making it more suitable for preserving long-range contextual dependencies and stable global geometric trends across distant local regions. Therefore, this branch mainly contributes to long-range context accumulation during sequential feature propagation. In contrast, the GRU branch adopts a more compact gated structure with update and reset gates, which can efficiently capture local feature variations between neighboring region tokens while filtering redundant sequential information. Therefore, the GRU branch mainly contributes to lightweight local feature variation modeling. By adaptively integrating these two branches with the current input token, AGF combines long-range context preservation, local feature variation modeling, and direct geometric information retention within a unified sequential interaction module. This complementary design is also consistent with the ablation results, where the complete AGF module achieves better performance than using a single branch alone.

#### 3.4.1. Gate-Scaled LSTM Branch

To enhance the flexibility of context accumulation during sequential propagation, we first introduce a Gate-Scaled LSTM branch. The network structure of the Gate-Scaled LSTM branch is illustrated by the red dashed box in Figure 1. For the *k*-th position in the point cloud sequence, given the current input token ${\hat{h}}_{k}$ and the hidden and cell states propagated from the previous sequence position $\left(h_{k-1}^{L},c_{k-1}^{L}\right)$, the combined representation is formulated as

(16) $$u_{k}=\left[{\hat{h}}_{k};h_{k-1}^{L}\right],$$

where [·;·] denotes channel-wise concatenation. Based on $u_{k}$, the input and forget gates are computed as

(17) $$i_{k}=\sigma \left((W_{i}u_{k})⊙\gamma \right),\;f_{k}=\sigma \left((W_{f}u_{k})⊙\gamma \right),$$

where $W_{i}$ and $W_{f}$ are learnable linear transformations, $\gamma \in R^{D_{L}}$ is a learnable gate scaling vector, σ(·) denotes the sigmoid activation function, and ⊙ denotes element-wise multiplication. The term “Gate-Scaled” indicates that the gate responses are adaptively scaled before sigmoid activation. Compared with standard LSTM gating, the scaling vector explicitly modulates the responses of the input and forget gates, enabling the state update process to better adapt to different structural contexts along the point cloud sequence. The candidate cell state and output gate are computed as

(18) $${\tilde{c}}_{k}=\tanh\left(W_{c}u_{k}\right),\;o_{k}=\sigma \left(W_{o}u_{k}\right),$$

where $W_{c}$ and $W_{o}$ are learnable linear mappings. The cell state and hidden state are then updated by

(19) $$c_{k}^{L}=f_{k}⊙c_{k-1}^{L}+i_{k}⊙{\tilde{c}}_{k},$$

(20) $$h_{k}^{L}=o_{k}⊙\tanh\left(c_{k}^{L}\right).$$

Here, $h_{k}^{L}\in R^{D_{L}}$ and $c_{k}^{L}\in R^{D_{L}}$ denote the hidden and cell states of the Gate-Scaled LSTM branch, respectively. In our implementation, $D_{L}=2D_{s}$, which provides a higher-capacity state space for long-range context modeling.

#### 3.4.2. Adaptive Gated Fusion

In parallel with the Gate-Scaled LSTM branch, a GRU branch is employed to capture complementary local variation patterns between adjacent sequence positions:

(21) $$h_{k}^{G}=\mathrm{GRU}\left({\hat{h}}_{k},h_{k-1}^{G}\right),$$

where $h_{k}^{G}\in R^{D_{s}}$ denotes the hidden state of the GRU branch. To make the outputs of the two sequential branches compatible for fusion, a dimension-aligned long-range context representation is obtained as

(22) $${\bar{h}}_{k}^{L}=\Pi \left(h_{k}^{L}\right),$$

where Π(·) denotes the dimension alignment operation. In our implementation, Π(·) selects the first $D_{s}$ channels of $h_{k}^{L}$.

The current input token, the aligned Gate-Scaled LSTM representation, and the GRU representation are concatenated to compute adaptive fusion weights:

(23) $$r_{k}=\left[{\hat{h}}_{k};{\bar{h}}_{k}^{L};h_{k}^{G}\right],$$

where $r_{k}\in R^{3D_{s}}$. In our implementation, the lightweight gating network is shared across all sequence positions and is implemented as a two-layer MLP:

(24) $$s_{k}=W_{2}\delta \left(W_{1}r_{k}+b_{1}\right)+b_{2},$$

where $W_{1}\in R^{D_{g}\times 3D_{s}}$, $W_{2}\in R^{3\times D_{g}}$. Here, $D_{g}$ denotes the hidden dimension of the gating network. δ(·) denotes the LeakyReLU activation function with a negative slope of 0.2. The output $s_{k}\in R^{3}$ represents the fusion logits for the three information sources. The adaptive fusion weights are then obtained by applying a softmax operation along the source dimension:

(25) $$\alpha_{k}=\mathrm{Softmax}\left(s_{k}\right),$$

where $\alpha_{k}=\left[\alpha_{k}^{\left(1\right)},\alpha_{k}^{\left(2\right)},\alpha_{k}^{\left(3\right)}\right]\in R^{3}$ denotes the fusion coefficients for the aligned Gate-Scaled LSTM representation, the GRU representation, and the current input token, respectively. The fused sequential output at the *k*-th sequence position is computed as

(26) $$z_{k}=\alpha_{k}^{\left(1\right)}{\bar{h}}_{k}^{L}+\alpha_{k}^{\left(2\right)}h_{k}^{G}+\alpha_{k}^{\left(3\right)}{\hat{h}}_{k}.$$

By stacking the fused outputs over all sequence positions, the final sequential representation is obtained as

(27) $$Z={\left\{z_{k}\right\}}_{k=1}^{M},\;Z\in R^{B\times M\times D_{s}}.$$

It is worth noting that GeoSeqNet does not rely on a strict ordering rule for raw points. Since sequential dependency learning is performed on geometry-enhanced local region representations rather than individual points, and geometric information is continuously preserved by GEM, the proposed framework reduces the influence of sequence ordering while maintaining geometric consistency during contextual learning. In addition to preserving geometric consistency, AGF also provides a more efficient alternative to self-attention-based contextual interaction. For an input sequence of length *M* and feature dimension $D_{s}$, self-attention-based methods typically require pairwise token interactions, leading to a computational complexity of $O(M^{2}D_{s})$ with respect to the sequence length. In contrast, the proposed AGF module performs contextual modeling through sequential feature propagation. Since the Gate-Scaled LSTM and GRU branches process the sequence step by step, the overall computational complexity of AGF can be approximated as $O(MD_{s}^{2})$ when the hidden-state dimension is comparable to $D_{s}$. The lightweight gating network introduces only a small additional cost and does not change the asymptotic complexity. Therefore, AGF grows linearly with the sequence length *M*, enabling efficient long-range contextual modeling while avoiding the quadratic token-to-token interaction overhead associated with self-attention mechanisms.

Overall, AGF integrates long-range context accumulation, adjacent-position variation perception, and direct input preservation into a unified sequential dependency learning framework. Compared with single-branch sequential modeling, the proposed design provides a more flexible mechanism for balancing different information sources during sequence propagation.Compared with self-attention-based contextual interaction, AGF captures cross-region contextual dependencies with lower computational complexity and memory overhead, making it more suitable for efficient and robust point cloud analysis.

## 4. Experiments

In this section, we first introduce the datasets and implementation details used in our experiments. We then compare the performance of GeoSeqNet with existing methods on several benchmark datasets. Finally, we conduct a series of ablation studies to validate the effectiveness of the proposed modules.

### 4.1. Experimental Settings

Datasets. To comprehensively evaluate the effectiveness of the proposed method under different spatial perception scenarios, experiments are conducted on three widely used benchmark datasets, including ModelNet40 [44], ScanObjectNN [45], and ShapeNetPart [46]. ModelNet40 is a synthetic CAD-based benchmark dataset for 3D object classification, containing 12,311 clean and uniformly sampled models from 40 object categories. Following the standard experimental protocol, 9843 samples are used for training and 2468 samples are used for testing. Since the dataset contains relatively regular geometric structures and limited sensing interference, it is mainly adopted to evaluate the global geometric representation capability of the proposed method under ideal spatial conditions. ScanObjectNN is a real-world scanned point cloud benchmark containing severe background clutter, occlusions, incomplete structures, and irregular sampling distributions. Following previous works, experiments are conducted on the PB_T50_RS split, which is considered one of the most challenging real-world point cloud benchmarks. Although ScanObjectNN is not a remote sensing dataset, its noisy and irregular scanning characteristics are highly consistent with practical LiDAR sensing scenarios in remote sensing applications. Therefore, this dataset is particularly suitable for evaluating the robustness and contextual propagation capability of the proposed method under complex sensing conditions. ShapeNetPart is a large-scale benchmark for point cloud part segmentation, containing 16 object categories and 50 part labels. The dataset provides fine-grained part-level annotations for evaluating local geometric decomposition capability and contextual structural understanding. Following the standard setting, each shape is uniformly sampled into 2048 points during both training and testing.

Implementation Details. All experiments were conducted using the PyTorch framework on two NVIDIA Tesla P40 GPUs (24 GB memory per GPU). Unless otherwise specified, all efficiency-related results, including inference speed and computational cost evaluations, were obtained under this hardware configuration. The AdamW optimizer is adopted with an initial learning rate of 0.001 and a batch size of 32. A cosine annealing scheduler is used for learning rate decay. During training, random rotation, scaling, and jittering are applied for data augmentation. GeoSeqNet is trained for 350 epochs on classification tasks and 300 epochs on part segmentation tasks. In all experiments, only the raw 3D coordinates (x,y,z) are used as network input. we follow standard input settings: 1024 points are used for ModelNet40, while 2048 points are sampled for both ScanObjectNN and ShapeNetPart. To ensure a fair comparison, all compared methods are evaluated under the same supervised learning setting. We follow standard benchmarks in point cloud classification and segmentation. Methods relying on large-scale pretraining or external datasets are not included in the comparison, as they adopt different training paradigms.

Evaluation Metrics. For classification tasks, overall accuracy (OA) and mean class accuracy (mAcc) are adopted as evaluation metrics. For part segmentation, mean Intersection-over-Union (mIoU) and instance-average Intersection-over-Union (Ins.mIoU) are reported. In addition to recognition performance, model efficiency is further evaluated using parameter counts and floating-point operations (FLOPs). These metrics are particularly important for large-scale spatial perception and remote sensing applications, where computational efficiency and robust contextual interaction are both critical requirements.

### 4.2. Classification Results on ModelNet40

Table 1 reports the classification results on ModelNet40. GeoSeqNet achieves an overall accuracy (OA) of 93.9% and a mean class accuracy (mAcc) of 91.8% while maintaining relatively low computational complexity. Compared with PointNet and PointNet++, GeoSeqNet achieves substantial performance improvements, demonstrating the effectiveness of geometry-aware contextual learning in extracting discriminative geometric representations. GeoSeqNet also achieves competitive performance relative to representative graph-based and Transformer-based methods, such as DGCNN and PCT, suggesting that preserving spatial geometric context during long-range contextual interaction facilitates more effective feature learning than relying solely on local aggregation or self-attention-based relation modeling. It should be noted that GeoSeqNet does not achieve the highest OA on ModelNet40. Some recent methods obtain comparable or higher classification accuracy on this benchmark. For example, DuGREAT achieves the highest OA of 94.6%, but its parameter size and computational cost reach 19.16M and 30 GFLOPs, respectively. In contrast, GeoSeqNet achieves competitive recognition performance with only 5.72M parameters and 2.0 GFLOPs. Therefore, the practical significance of GeoSeqNet on ModelNet40 does not lie in absolute accuracy superiority, but in achieving a favorable efficiency–accuracy trade-off. This result indicates that when both classification accuracy and computational cost are considered, GeoSeqNet can provide a relatively compact and efficient solution.

### 4.3. Classification Results on ScanObjectNN

Unlike ModelNet40, ScanObjectNN is constructed from real-world scanned objects and contains severe sensing degradation, including noisy observations, background clutter, partial occlusion, and irregular point distributions. Therefore, performance on this benchmark more directly reflects the robustness and generalization capability of point cloud models under complex real-world sensing conditions. The quantitative comparisons on ScanObjectNN are summarized in Table 2. To provide a more stable evaluation of GeoSeqNet, we conducted three independent runs under the same experimental setting. The obtained OA values are 88.81%, 88.74%, and 88.76%, resulting in an average OA of ((88.77±0.04)%), which is reported as 88.8% in Table 2. GeoSeqNet achieves the best overall performance, obtaining an OA of 88.8% and an mAcc of 88.0%, while maintaining a relatively low computational cost of 2.0 GFLOPs and 5.72M parameters. Compared with Transformer-based methods such as PCT and PointConT, GeoSeqNet consistently achieves higher recognition accuracy with fewer parameters and lower computational overhead. This indicates that preserving spatial geometric context during long-range contextual interaction is more effective than relying solely on self-attention-based global relation modeling under noisy and irregular point cloud conditions. GeoSeqNet also outperforms several recent state-space-based methods, including PointMamba, SI-Mamba, and Mamba3D. Although these approaches improve computational efficiency through sequential or state-space modeling mechanisms, their ability to maintain stable geometric representations under sensing degradation remains limited when explicit geometric context is insufficiently preserved during feature interaction. In contrast, GeoSeqNet jointly integrates local neighborhood refinement, coordinate-aware geometric context preservation, and adaptive contextual propagation, resulting in more stable geometric representation learning under challenging real-world sensing environments.

**Figure 2:**
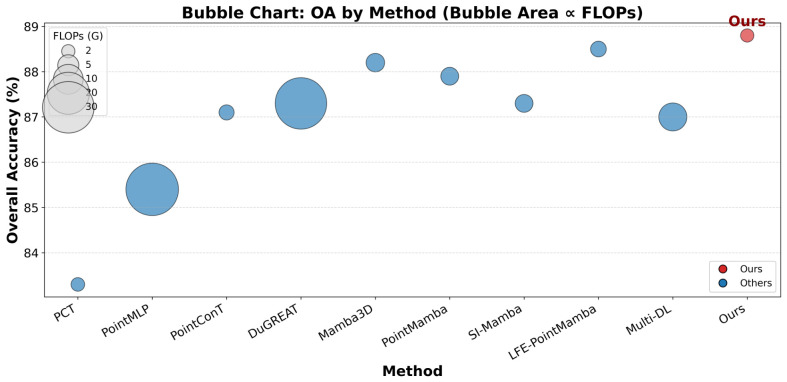
Comparison of overall accuracy (OA) and computational complexity of different point cloud classification methods on the ScanObjectNN dataset. Bubble area is proportional to FLOPs. GeoSeqNet achieves the highest OA with low computational complexity, demonstrating a favorable accuracy–efficiency trade-off.

To further illustrate the trade-off between recognition accuracy and computational efficiency, Figure 2 presents a bubble chart comparison on ScanObjectNN, where the bubble area is proportional to FLOPs. It can be observed that several methods with substantially larger computational costs, such as PointMLP and DuGREAT, still fail to achieve better recognition performance than GeoSeqNet. This phenomenon suggests that simply increasing model complexity or global interaction capacity does not necessarily improve robustness under noisy observations, incomplete geometry, and irregular point distributions. In contrast, GeoSeqNet is located in the upper region of the accuracy–efficiency space with a relatively small bubble size, demonstrating that the proposed geometry-aware contextual learning strategy achieves a more favorable balance between robustness and computational efficiency. These empirical observations are also consistent with the theoretical complexity analysis presented in the method section, further supporting the efficiency advantage of the proposed AGF module. Overall, the results in Table 2 and Figure 2 consistently demonstrate that GeoSeqNet not only achieves competitive classification performance but also maintains superior computational efficiency under complex real-world sensing conditions. We attribute this advantage to the collaborative interaction among three complementary components: ELO suppresses unreliable neighborhood responses caused by irregular sampling and sensing noise, GEM continuously preserves spatial geometric context during long-range contextual interaction, and AGF enables stable contextual propagation with relatively low computational overhead.

**Figure 3:**
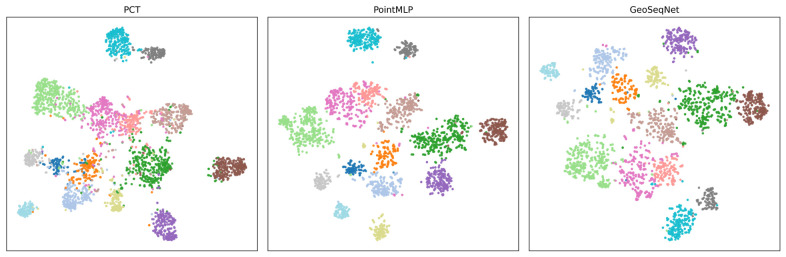
Comparison of learned feature embeddings visualized by t-SNE on the ScanObjectNN dataset, where different colors represent different object categories.

Finally, to analyze the learned feature representations of the model, we introduce t-SNE visualization of feature embeddings. As shown in Figure 3, it can be observed that PCT and PointMLP exhibit a certain degree of class overlap in the feature space, whereas GeoSeqNet presents a more clearly separated class structure with significantly reduced inter-class mixing. This result indicates that GeoSeqNet learns more discriminative feature representations. Its geometry-aware encoding and long-range contextual modeling contribute to improved separability and discriminability in the feature space, thereby enhancing overall model performance.

### 4.4. Part Segmentation Results on ShapeNetPart

**Figure 4:**
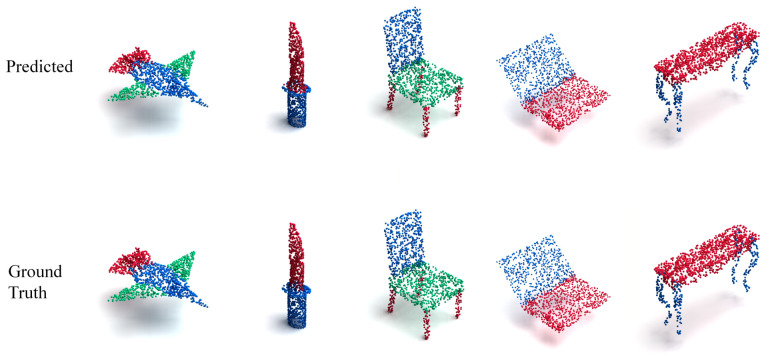
Visualization results for part segmentation using GeoSeqNet. Points are marked with different colors based on the parts to which they belong. The first row shows the model’s predicted results, and the second row displays the Ground Truth.

The part segmentation results on ShapeNetPart are summarized in Table 3. GeoSeqNet achieves an instance mIoU of 86.0%, demonstrating strong competitiveness against existing methods. Compared with recent strong baselines such as PointMLP and DSACNN, GeoSeqNet further achieves consistent performance improvements, indicating that geometry-aware contextual learning remains beneficial for fine-grained structural understanding. From the category-wise results, GeoSeqNet achieves the best performance on bag, cap, guitar, knife, and mug, while obtaining tied-best performance on laptop and skateboard. These categories usually require joint modeling of local geometric details and long-range structural consistency. The experimental results indicate that GeoSeqNet is capable of maintaining stable contextual interaction across spatially separated structural components. Meanwhile, GeoSeqNet does not yet achieve the best results on categories with more drastic shape variations or highly slender structures, such as rocket and lamp. This suggests that further improvements in geometric sensitivity and adaptive contextual interaction remain possible for extremely complex geometric structures. Qualitative visualization results are shown in Figure 4. GeoSeqNet produces structurally consistent segmentation boundaries and demonstrates robust part discrimination ability across different object categories.

### 4.5. Ablation Study

To further validate the effectiveness of the proposed framework, extensive ablation studies are conducted on ScanObjectNN. Compared with ModelNet40 and ShapeNetPart, ScanObjectNN contains more severe sensing degradation, including noisy observations, background clutter, partial occlusions, and irregular point distributions, thereby providing a more challenging benchmark for evaluating module effectiveness. The increased geometric complexity and sensing uncertainty in ScanObjectNN enable a more rigorous assessment of the individual contributions of ELO, GEM, and AGF than relatively clean benchmark datasets.

#### 4.5.1. Overall Module Ablation

Table 4 presents the overall module ablation analysis on ScanObjectNN. The pure MLP baseline achieves an OA of 81.5%, indicating that feature transformation alone is insufficient for robust geometric representation learning under complex sensing conditions. Introducing ELO improves the OA to 86.6%, demonstrating that explicitly refining neighborhood responses helps suppress unreliable local geometric interference caused by irregular sampling and sensing noise. Incorporating GEM further improves the OA to 86.9%, suggesting that preserving coordinate-aware spatial context is beneficial for stabilizing geometric representation learning during long-range contextual interaction. Among all individual modules, AGF achieves the most significant performance gain, increasing the OA to 87.9%. This indicates that long-range contextual interaction plays an important role in point cloud understanding under noisy and irregular real-world sensing conditions. Further combining ELO or GEM with AGF consistently improves performance. Specifically, ELO+AGF and GEM+AGF achieve 88.2% and 88.4% OA, respectively, and the complete GeoSeqNet achieves the best performance of 88.8%. These results show that local geometry refinement, geometric context preservation, and adaptive long-range contextual interaction provide complementary benefits for robust point cloud understanding.

To further examine the interaction between ELO and GEM, we analyze their combination with and without AGF. When AGF is absent, ELO+GEM achieves an OA of 87.1%, which is only slightly higher than ELO alone (86.6%) and GEM alone (86.9%). This indicates that directly combining local geometric refinement and geometric encoding brings limited additional benefit without long-range contextual propagation. In contrast, when AGF is introduced, both ELO and GEM further improve the model performance. ELO+AGF improves the OA to 88.2%, while GEM+AGF achieves a higher OA of 88.4%. This suggests that the contribution of GEM becomes more evident in the presence of long-range contextual interaction, since the coordinate-aware geometric information preserved by GEM can be more effectively utilized during cross-region feature propagation. Meanwhile, ELO provides refined local geometric features that can support more reliable AGF-based contextual modeling.

In addition, we report the additional parameter cost introduced by each module in Table 5. ELO and GEM introduce only 0.0682M and 0.0090M additional parameters, respectively, while AGF accounts for the major parameter increase due to its sequential contextual modeling structure. Combined with the ablation results, these parameter statistics show that ELO, GEM, and AGF contribute to performance improvement with reasonable module-level parameter costs. Overall, the results indicate that the three modules are complementary: ELO focuses on local geometric enhancement, GEM preserves geometric context, and AGF performs long-range contextual interaction.

#### 4.5.2. Ablation on Contextual Interaction Components

Table 6 compares different contextual interaction strategies. Using only the GSLSTM branch achieves better performance than using only GRU, indicating that stable long-range contextual propagation plays an important role in robust geometric representation learning. Compared with the more locally oriented contextual updating behavior of GRU, GSLSTM maintains more continuous contextual propagation across larger spatial regions, which is beneficial for preserving cross-region geometric consistency. Further combining GSLSTM and GRU leads to additional performance improvements, suggesting that long-range contextual consistency and local structural variation provide complementary information.

In addition, we further compare the proposed long-range contextual interaction strategy with conventional LSTM and BiLSTM architectures. As shown in Table 7, the proposed GSLSTM achieves more stable and superior performance. This indicates that conventional fixed gating mechanisms are less effective at dynamically adjusting contextual propagation strength according to different geometric structures in complex point cloud scenes. By contrast, the proposed gate scaling strategy enables more flexible long-range contextual interaction through learnable gate scaling vectors, making it more suitable for complex 3D geometric structure modeling.

Finally, we conduct an ablation study on the adaptive gated fusion mechanism. As shown in Table 8, equal-weight fusion performs noticeably worse than adaptive fusion. This suggests that different spatial regions exhibit varying dependencies on local geometric details and global contextual information. The adaptive gated fusion mechanism dynamically adjusts the importance of different feature branches according to the current geometric context, thereby enabling more stable and robust contextual interaction.

#### 4.5.3. Ablation on Spatial Weighting in Enhanced Local Operator

Table 9 evaluates the effectiveness of the spatial weighting mechanism in ELO. Removing spatial weighting leads to a noticeable performance drop, indicating that selective neighborhood response refinement is important for suppressing unreliable geometric responses under irregular point distributions and noisy observations. This is mainly because neighboring points in real-world point clouds often exhibit inconsistent geometric quality due to sensing noise, density variation, and incomplete observations. Directly aggregating all neighborhood features with equal importance may therefore introduce unstable local responses. By contrast, the proposed spatial weighting mechanism dynamically adjusts neighborhood contributions according to geometric relevance, enabling the network to emphasize structurally informative regions while suppressing unreliable geometric interference. The results further demonstrate that geometry-aware neighborhood refinement substantially improves robust point cloud understanding under complex sensing conditions.

#### 4.5.4. Effect of Sequence Ordering Strategies

To investigate the influence of sequence ordering on GeoSeqNet, we evaluate several sequence construction strategies, including sorting along the *x*-axes, *y*-axes, and *z*-axes, random ordering, and the default sequence configuration. The results are summarized in Table 10. It can be observed that GeoSeqNet achieves consistently strong performance under different axis-based ordering strategies. The performance variation among *x*-axis, *y*-axis, and *z*-axis ordering remains within 0.1%, indicating that the proposed framework is not sensitive to a specific geometric ordering strategy. Although random ordering leads to a slight decrease in performance, the degradation is limited, suggesting that GeoSeqNet still maintains effective contextual learning capability without relying on a strict sequence arrangement. We attribute this robustness to the geometry-aware design of the proposed framework. Specifically, ELO extracts stable local geometric representations, while GEM continuously preserves spatial geometric context during feature interaction. Consequently, sequential learning is performed on geometry-enhanced local region representations rather than arbitrarily ordered raw points, making the model less sensitive to sequence ordering while preserving effective long-range contextual dependencies.

#### 4.5.5. Effect of Geometric Encoding and Fusion Strategy

To further analyze the rationality of the GEM design, we conduct additional ablation experiments on different geometric encoding methods and feature fusion strategies. Since GEM aims to reintroduce coordinate-aware geometric context during long-range feature interaction, the choice of coordinate encoder and fusion strategy directly affects the effectiveness of the geometric prior. Table 11 presents the experimental results of different geometric encoding methods. Compared with sinusoidal positional encoding and learnable Fourier feature encoding, the MLP-based encoding adopted in this work achieves the best performance. This result indicates that directly introducing frequency-based coordinate representations is not necessarily the most suitable choice for GEM. Sinusoidal positional encoding relies on fixed frequency bases and has limited adaptability to different point cloud distributions. Although learnable Fourier feature encoding introduces trainable frequency projections and provides stronger representational flexibility, it may also emphasize certain high-frequency coordinate variations, which are not necessarily beneficial for region-level geometric context modeling. In contrast, the two-layer MLP encoder can learn compact nonlinear geometric representations from raw coordinates, adaptively encoding coordinate information according to the training objective while avoiding excessive positional complexity. Table 12 further compares the influence of different fusion strategies in GEM. Additive fusion with learnable weights and gated fusion achieve OA values of 88.2% and 88.6%, respectively. The performance of these two fusion strategies is still slightly lower than that of the concatenation-based fusion adopted in this work. A possible reason is that additive fusion directly adds semantic features and geometric features after projecting them into the same feature space, which may weaken their independent complementary information. Although gated fusion can adaptively control the introduction of geometric features, it may also suppress some useful geometric cues at an early stage. In contrast, concatenation-based fusion does not force the two types of features to be immediately mixed. Instead, it preserves the original feature representation and the geometric encoding representation before subsequent feature transformation, enabling the subsequent network layers to exploit their complementary information more flexibly. Therefore, this work adopts the two-layer MLP encoder and concatenation-based fusion as the final design of GEM.

### 4.6. Robustness Analysis

**Figure 5:**
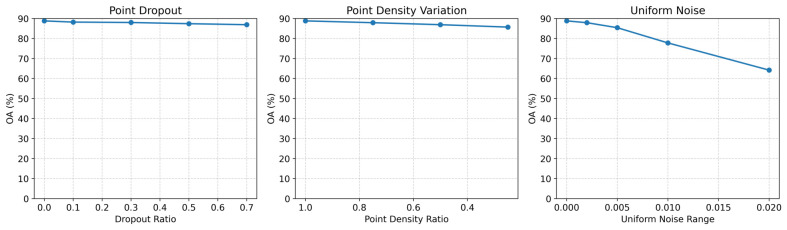
Robustness analysis of the proposed method under different point cloud corruption settings on the ScanObjectNN dataset, including point dropout, density variation, and uniform noise perturbation. The curves illustrate the performance degradation trends as the severity of each perturbation increases.

To evaluate the robustness of the proposed method, we conduct experiments on the ScanObjectNN dataset under three representative point cloud corruption settings, including point dropout, density variation, and uniform noise perturbation. The results are illustrated in Figure 5. A clear distinction can be observed between structural perturbations (point dropout and density variation) and coordinate-level perturbations (uniform noise). Under structural corruption, the model exhibits only slight performance degradation. Specifically, even when 70% of points are removed, the accuracy decreases marginally from 88.8% to 86.9%. Similarly, under density variation, the performance decreases smoothly, and the model still achieves 85.7% accuracy when only 25% of points are retained, indicating strong robustness to incomplete and sparse geometric observations. In contrast, uniform noise leads to a more pronounced performance drop, where accuracy decreases from 88.8% to 64.2% as the noise level increases. This suggests that coordinate-level perturbations have a significantly stronger impact on feature representation than structural corruption. This behavior can be attributed to the fact that the proposed method relies on local neighborhood construction and hierarchical geometric feature encoding, which are sensitive to accurate relative spatial relationships. Structural perturbations preserve the overall geometric topology, enabling stable feature aggregation. However, uniform noise directly disrupts coordinate consistency, leading to incorrect neighborhood construction and degraded geometric representation. Overall, the proposed method demonstrates strong robustness to structural corruption, while being more sensitive to coordinate-level noise. This indicates that the model effectively models geometric structure consistency, but its robustness to high-frequency coordinate perturbations remains limited.

## 5. Conclusions

In this work, we propose GeoSeqNet, a geometry-aware contextual learning framework for efficient and robust 3D point cloud analysis. Different from methods that mainly rely on fixed local aggregation or computationally expensive global interaction mechanisms, GeoSeqNet aims to preserve spatial geometric context during long-range contextual interaction. Specifically, the proposed framework integrates an Enhanced Local Operator (ELO), a Geometric Encoding Module (GEM), and an Adaptive Gate Fusion (AGF) module within a unified architecture. Through the collaboration of local geometry refinement, coordinate-aware geometric context preservation, and adaptive contextual propagation, GeoSeqNet effectively captures both fine-grained local structures and cross-region dependencies while maintaining relatively low computational overhead. Extensive experiments on ModelNet40, ScanObjectNN, and ShapeNetPart demonstrate the effectiveness of the proposed method. GeoSeqNet achieves competitive performance with a favorable efficiency–accuracy trade-off. In particular, its strong performance on the real-world ScanObjectNN benchmark indicates improved robustness under incomplete geometry, background interference, and irregular sampling conditions. These results suggest that preserving spatial geometric context during long-range interaction is beneficial for robust point cloud understanding in complex real-world sensing environments. Beyond classification and segmentation, this work also highlights the potential of geometry-aware contextual propagation for point cloud representation learning. Compared with purely attention-based architectures, the combination of explicit geometric modeling and lightweight long-range contextual learning provides a suitable inductive bias for complex 3D geometric data. In future work, we will further investigate more robust neighborhood construction and geometry-aware contextual interaction strategies under sparse observations and severe sensing degradation. Extending GeoSeqNet to dynamic point cloud perception, large-scale sensing applications, and pretraining-based representation learning also remains an important direction for future research.

## Figures and Tables

**Table 1:** Classification results on ModelNet40 dataset.

Method	mAcc (%)	OA (%)	Param. (M)	Test Speed	Flops (GFlops)
PointNet [5]	86.2	89.2	3.5	-	**0.5**
PointNet++ [6]	-	91.9	1.7	425	1.7
PointCNN [7]	88.1	92.5	**0.6**	-	0.9
DGCNN [20]	90.2	92.9	1.8	**503**	-
KPConv [19]	-	92.9	-	-	-
PCT [12]	-	93.2	2.88	325	2.17
PointMLP [23]	91.4	93.6	12.6	154	15.73
PointConT(no voting) [13]	-	92.8	14.66	143	2.59
DuGREAT [15]	91.4	**94.6**	19.16	-	30
PointMamba [29]	-	92.8	12.3	-	3.6
Mamba3D [30]	-	93.4	16.9	43	3.25
SI-Mamba [32]	-	92.7	12.3	-	3.6
Multi-DL [47]	91.0	93.3	8.73	441	8.9
Ours	**91.8**	93.9	5.72	138	2.0

Note: Test Speed = the number of samples/test time(s). The “-” symbol indicates that this metric was not provided in the original paper or code. Bold and underlined values indicate the best and second-best results, respectively.

**Table 2:** Classification results on ScanObjectNN dataset.

Method	mAcc (%)	OA (%)	Param. (M)	Test Speed	Flops (GFlops)
PointNet [5]	63.4	68.2	3.5	-	**0.5**
PointNet++ [6]	75.4	77.9	1.7	-	1.7
PointCNN [7]	75.1	78.5	**0.6**	-	0.9
DGCNN [20]	73.6	78.1	1.8	-	-
PCT [12]	80.5	83.3	2.88	158	2.17
PointMLP [23]	83.9	85.4	12.6	153	15.73
DuGREAT [15]	85.5	87.3	19.16	-	30
PointConT(no voting) [13]	-	87.1	14.66	122	2.59
PointMamba [29]	-	87.9	12.3	-	3.6
Mamba3D [30]	-	88.2	16.9	43	3.9
SI-Mamba [32]	-	87.3	12.3	-	3.6
LFE-PointMamba [31]	-	88.5	9.0	-	2.8
Multi-DL [47]	85.5	87.0	8.73	**264**	8.9
Ours	**88.0**	**88.8**	5.72	161	2.0

Note: Test Speed = the number of samples/test time(s). The “-” symbol indicates that this metric was not provided in the original paper or code. Bold and underlined values indicate the best and second-best results, respectively.

**Table 3:** The experimental results for part segmentation on the ShapeNet Parts dataset were compared.

Method	Ins IoU	Aero	Bag	Cap	Car	Chair	Ear Phone	Guitar	Knife	Lamp	Laptop	Motor Bike	Mug	Pistol	Rocket	Skate Board	Table
Pointnet [5]	83.7	83.4	78.7	82.5	74.9	89.6	73.0	91.5	85.9	80.8	95.3	65.2	93.0	81.2	57.9	72.8	80.6
Pointnet++ [6]	85.1	82.4	79.0	87.7	77.3	90.8	71.8	91.0	85.9	83.7	95.3	71.6	94.1	81.3	58.7	76.4	82.6
DGCNN [20]	85.2	**84.0**	83.4	86.7	77.8	90.6	74.7	91.2	87.5	82.8	95.7	66.3	94.9	81.1	63.5	74.5	82.6
PCNN [48]	85.1	82.4	83.8	88.2	**80.5**	**91.1**	76.2	91.9	87.6	**84.9**	95.8	70.7	95.3	82.4	63.6	72.7	82.8
P2Sequence [49]	85.2	82.6	81.8	87.5	77.3	90.8	77.1	91.1	86.9	83.9	95.7	70.8	94.6	79.3	58.1	75.2	82.8
SpiderCNN [10]	85.3	83.5	81.0	87.2	77.5	90.7	76.8	91.1	87.3	83.3	95.8	70.2	93.5	82.7	59.7	75.8	82.8
PCT [12]	84.8	80.6	82.9	**91.1**	77.0	89.8	78.5	91.3	87.3	80.6	95.8	68.8	93.7	81.9	60.9	78.7	**83.5**
PointMLP [23]	85.9	83.3	83.2	89.1	79.7	90.0	**80.3**	91.8	88.1	82.8	96.2	**76.9**	94.5	83.7	**66.3**	**82.2**	**83.5**
PointMamba [29]	85.8	-	-	-	-	-	-	-	-	-	-	-	-	-	-	-	-
Mamba3D [30]	85.7	-	-	-	-	-	-	-	-	-	-	-	-	-	-	-	-
Ours	**86.0**	82.8	**85.2**	90.2	79.2	90.0	79.3	**92.2**	**89.1**	80.4	**96.3**	73.4	**95.9**	**83.9**	62.5	**82.2**	83.2

Note: The “-” symbol indicates that this metric was not provided in the original paper or code. Bold and underlined values indicate the best and second-best results, respectively.

**Table 4:** Overall module ablation analysis of GeoSeqNet on the ScanObjectNN benchmark.

Setting	ELO	GEM	AGF	mAcc (%)	OA (%)
Baseline (Pure MLP)	✗	✗	✗	78.8	81.5
+ ELO	✓	✗	✗	85.5	86.6
+ GEM	✗	✓	✗	85.7	86.9
+ AGF	✗	✗	✓	86.9	87.9
+ ELO + GEM	✓	✓	✗	85.9	87.1
+ ELO + AGF	✓	✗	✓	87.1	88.2
+ GEM + AGF	✗	✓	✓	87.6	88.4
Full GeoSeqNet	✓	✓	✓	**88.0**	**88.8**

Note: ✓ and ✗ indicate whether the corresponding module is enabled or disabled. Bold values indicate the best results.

**Table 5:** Additional parameter cost introduced by each module in GeoSeqNet.

Module	Param. (M)
ELO	0.0682
GEM	0.0090
AGF	4.5778

**Table 6:** Comparison of different sequential modeling components on the ScanObjectNN dataset.

Setting	mAcc (%)	OA (%)
GSLSTM	86.4	87.6
GRU	84.8	87.0
GSLSTM + GRU	87.2	88.4
Adaptive Gate Fusion Block	**88.0**	**88.8**

Note: Bold values indicate the best results.

**Table 7:** Comparison of different long-range contextual interaction strategies on ScanObjectNN.

Setting	mAcc (%)	OA (%)
LSTM	85.9	87.1
BiLSTM	86.2	87.4
GSLSTM	**86.4**	**87.6**

Note: Bold values indicate the best results.

**Table 8:** Ablation on fusion weighting strategy in Adaptive Gate Fusion Block on ScanObjectNN.

Setting	mAcc (%)	OA (%)
Fixed weights (1:1)	87.1	88.5
Adaptive fusion	**88.0**	**88.8**

Note: Bold values indicate the best results.

**Table 9:** Comparison between Local Operator and Enhanced Local Operator on ScanObjectNN.

Setting	mAcc (%)	OA (%)
Local Operator (*w*/*o* spatial weighting)	87.6	88.4
ElO	**88.0**	**88.8**

Note: Bold values indicate the best results.

**Table 10:** Influence of Sequence Ordering Strategies on Model Performance.

Sequence Strategy	mAcc (%)	OA (%)
*x*-axis	88.79	**88.85**
*y*-axis	87.93	88.80
*z*-axis	88.00	88.78
Random Order	87.43	88.52
Original Order	**88.01**	88.84

Note: Bold values indicate the best results.

**Table 11:** Effect of different geometric encoding strategies in GEM.

Encoding Strategy	mAcc (%)	OA (%)
Sinusoidal positional encoding	86.1	87.4
Learnable Fourier Features	87.2	88.1
MLP	**88.0**	**88.8**

Note: Bold values indicate the best results.

**Table 12:** Effect of different feature fusion strategies in GEM.

Fusion Strategy	mAcc (%)	OA (%)
Gated fusion	87.7	88.6
Additive fusion with learnable weights	87.1	88.2
Concatenation	88.0	88.8

Note: Bold values indicate the best results.

## Data Availability

The original contributions presented in the study are included in the article, further inquiries can be directed to the corresponding author.
